# ICTV Virus Taxonomy Profile: *Hypoviridae*

**DOI:** 10.1099/jgv.0.001055

**Published:** 2018-03-28

**Authors:** Nobuhiro Suzuki, Said A. Ghabrial, Kook-Hyung Kim, Michael Pearson, Shin-Yi L. Marzano, Hajime Yaegashi, Jiatao Xie, Lihua Guo, Hideki Kondo, Igor Koloniuk, Bradley I. Hillman, ICTV Report Consortium

**Affiliations:** ^1^​ Institute of Plant Science and Resources, Okayama University, Kurashiki, Okayama 710-0046, Japan; ^2^​ Plant Pathology Department, University of Kentucky, Lexington, KY 40504, USA; ^3^​ Department of Agricultural Biotechnology and College of Agriculture and Life Sciences, Seoul National University, Seoul 08826, Republic of Korea; ^4^​ School of Biological Sciences, The University of Auckland, Private Bag 92019, Auckland, New Zealand; ^5^​ Department of Biology and Microbiology, South Dakota State University, Brookings, SD 57007, USA; ^6^​ Department of Agronomy, Horticulture, and Plant Science, South Dakota State University, Brookings, SD 57007, USA; ^7^​ Division of Apple Research, Institute of Fruit Tree and Tea Science, National Agriculture and Food Research Organization (NARO), Morioka, Japan; ^8^​ Plant Pathology Department, College of Plant Science and Technology, Huazhong Agricultural University, Wuhan 430070, Hubei Province, PR China; ^9^​ State Key Laboratory for Biology of Plant Disease and Insect Pests, Institute of Plant Protection, Chinese Academy of Agricultural Sciences, Beijing 100193, PR China; ^10^​ Department of Plant Virology, Institute of Plant Molecular Biology, Biology Centre of the Czech Academy of Sciences, v.v.i., Branišovská 31, 370 05 České Budějovice, Czech Republic; ^11^​ Department of Plant Biology and Pathology, Rutgers University, New Brunswick, NJ 08901, USA

**Keywords:** *Hypoviridae*, ICTV Report, Taxonomy

## Abstract

The *Hypoviridae*, comprising one genus, *Hypovirus*, is a family of capsidless viruses with positive-sense, ssRNA genomes of 9.1–12.7 kb that possess either a single large ORF or two ORFs. The ORFs appear to be translated from genomic RNA by non-canonical mechanisms, i.e. internal ribosome entry site-mediated and stop/restart translation. Hypoviruses have been detected in ascomycetous or basidiomycetous filamentous fungi, and are considered to be replicated in host Golgi-derived, lipid vesicles that contain their dsRNA as a replicative form. Some hypoviruses induce hypovirulence to host fungi, while others do not. This is a summary of the current ICTV report on the taxonomy of the *Hypoviridae*, which is available at www.ictv.global/report/hypoviridae.

## Virion

**Fig. 1.**
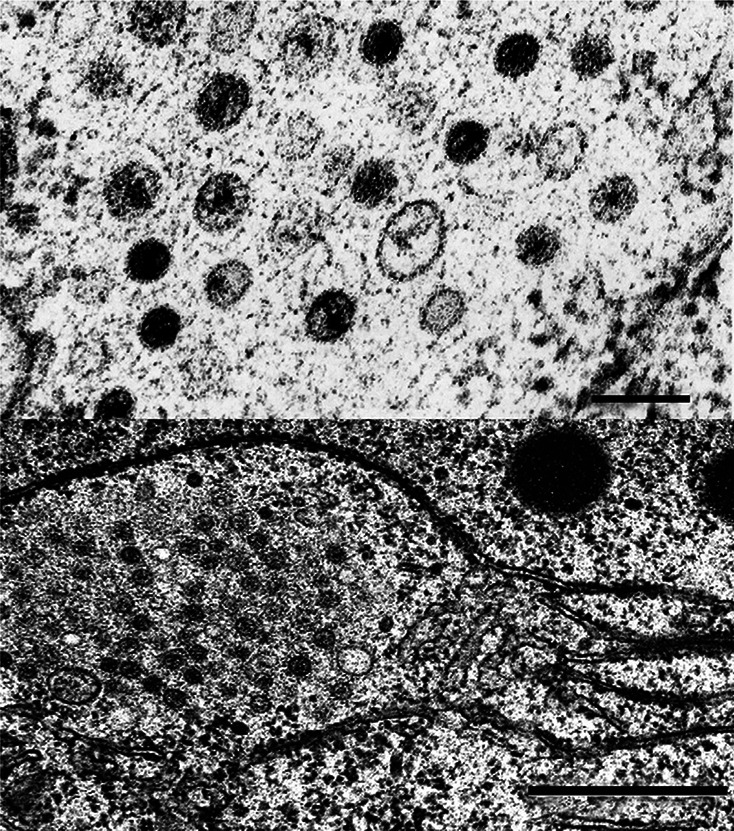
Thin sections showing (top) vesicles in fungal tissue; (bottom) vesicle aggregate in fungal tissue surrounded by rough endoplasmic reticulum. Bar, 100 nm. Reproduced with permssion from reference [1].

No true virions are associated with members of the family *Hypoviridae*. Pleomorphic vesicles 50–80 nm in diameter [1], devoid of any detectable viral structural proteins but containing replicative form dsRNA and polymerase activity [2], are the only virus-associated particles that can be isolated from infected fungal tissue (Table 1, Fig. 1).

**Table 1.** Characteristics of the family *Hypoviridae*

Typical member:	Cryphonectria hypovirus 1 strain EP713 (M57938), species *Cryphonectria hypovirus 1*, genus *Hypovirus*
Virion	Capsidless virus unable to form rigid particles
Genome	9.1–12.7 kb of linear, positive-sense, non-segmented RNA
Replication	Replication (synthesis of complementary RNA) and transcription (synthesis of genomic RNA) occur cytoplasmically in Golgi-derived membraneous vesicles
Translation	Directly from bi- or monocistronic genomic RNA containing a possible internal ribosomal entry site at the 5′-non-coding region
Host range	Fungi
Taxonomy	One genus including four species

## Genome

**Fig. 2.**
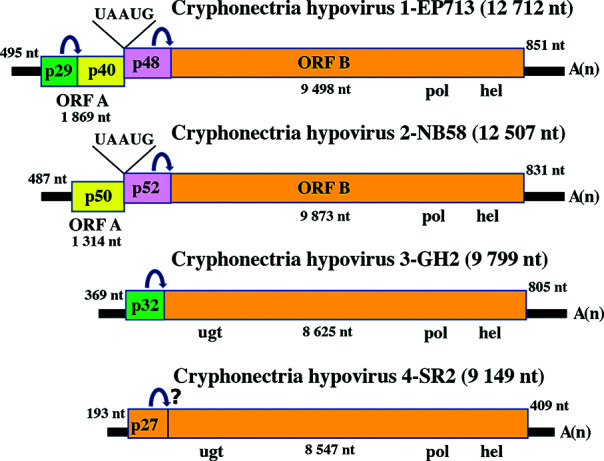
Genome organization of four members of the family *Hypoviridae*. Arrows represent known or putative (Cryphonectria hypovirus 4) sites of autoproteolysis. The abbreviations pol, hel and ugt refer to the RNA-dependent RNA polymerase, RNA helicase and UDP-glucose/sterol glucosyltransferase domains, respectively.

Hypovirus genomes range from 9.1 to 12.7 kb excluding a 3′-poly(A) tail of 20–30 nt, and possess one or two ORFs (Fig. 2) [3] flanked by relatively long 5′- and 3′-terminal non-coding regions. Translational initiation for the first ORF on the genomic RNA is mediated by an internal ribosome entry site in the 5′-non-coding region extending to the coding domain in the case of Cryphonectria hypovirus 1. For hypoviruses with a two-ORF genome organization, the stop/restart translation mechanism is involved in the translation of downstream ORFs in which the pentamer, UAAUG (Fig. 2), plays a critical role [4]. Many hypoviruses have shorter-than-full-length, internally-deleted, defective interfering and defective replicative form dsRNA molecules; others have replicative forms of satellite-like RNAs [5, 6]. The host RNA silencing pathway has been reported to promote defective interfering RNA production [7]. No function has been ascribed to any ancillary dsRNA.

## Replication

Positive- and negative-strand viral RNA synthesis is believed to occur cytoplasmically in host-derived lipid vesicles that contain linear dsRNA, regarded as the replicative form of hypoviral genomic positive-sense ssRNA. The polymerase associated with vesicles transcribes ssRNA molecules *in vitro* that correspond in size to full-length dsRNA. Approximately 80 % of the polymerase products *in vitro* are of positive-sense. Except for the p50 of Cryphonectria hypovirus 2, hypovirus proteins are synthesized as part of a polyprotein that is autocatalytically cleaved by viral proteases such as p29 and p48 (Cryphonectria hypovirus 1) and p52 (Cryphonectria hypovirus 2). Smaller proteins encoded by the 3′-proximal ORF of Cryphonectria hypovirus 1 have been identified in the vesicle-associated polymerase complex, suggesting extensive processing of the ORF B-encoded polyprotein *in vivo* by unknown viral or host proteases. Cryphonectria hypovirus 1 p29 enhances virus replication *in cis* and *in trans* possibly by suppressing antiviral RNA silencing [7]. The p48 protein encoded by Cryphonectria hypovirus 1 ORF B is required for initiation but not maintenance of viral RNA replication [8].

## Taxonomy

The genus *Hypovirus* includes four species: *Cryphonectria hypovirus 1*, *Cryphonectria hypovirus 2*, *Cryphonectria hypovirus 3* and *Cryphonectria hypovirus 4* [3]. Unclassified hypoviruses include Sclerotinia sclerotiorum hypovirus 2 [9].

## Resources

Full ICTV Online (10th) Report: www.ictv.global/report/hypoviridae.
